# Inference of complex biological networks: distinguishability issues and optimization-based solutions

**DOI:** 10.1186/1752-0509-5-177

**Published:** 2011-10-28

**Authors:** Gábor Szederkényi, Julio R Banga, Antonio A Alonso

**Affiliations:** 1 (Bio)Process Engineering Group, IIM-CSIC, Spanish National Research Council, C/Eduardo Cabello, 6, 36208 Vigo, Spain; 2 Process Control Research Group, MTA SZTAKI, Kende u. 13-17, H-1111 Budapest, Hungary

## Abstract

**Background:**

The inference of biological networks from high-throughput data has received huge attention during the last decade and can be considered an important problem class in systems biology. However, it has been recognized that reliable network inference remains an unsolved problem. Most authors have identified lack of data and deficiencies in the inference algorithms as the main reasons for this situation.

**Results:**

We claim that another major difficulty for solving these inference problems is the frequent lack of uniqueness of many of these networks, especially when prior assumptions have not been taken properly into account. Our contributions aid the distinguishability analysis of chemical reaction network (CRN) models with mass action dynamics. The novel methods are based on linear programming (LP), therefore they allow the efficient analysis of CRNs containing several hundred complexes and reactions. Using these new tools and also previously published ones to obtain the network structure of biological systems from the literature, we find that, often, a unique topology cannot be determined, even if the structure of the corresponding mathematical model is assumed to be known and all dynamical variables are measurable. In other words, certain mechanisms may remain undetected (or they are falsely detected) while the inferred model is fully consistent with the measured data. It is also shown that sparsity enforcing approaches for determining 'true' reaction structures are generally not enough without additional prior information.

**Conclusions:**

The inference of biological networks can be an extremely challenging problem even in the utopian case of perfect experimental information. Unfortunately, the practical situation is often more complex than that, since the measurements are typically incomplete, noisy and sometimes dynamically not rich enough, introducing further obstacles to the structure/parameter estimation process. In this paper, we show how the structural uniqueness and identifiability of the models can be guaranteed by carefully adding extra constraints, and that these important properties can be checked through appropriate computation methods.

## Background

During the last decade, the wide availability of high-throughput biological data has made it possible to produce new knowledge via a systems biology approach [1-3]. The inference of biochemical networks (i.e. the mathematical mapping of the molecular interactions in the cell) is therefore a question of key importance in the field. During the last decade, many methods have been developed to solve the network-inference (sometimes called reverse-engineering [4]) problems arising in e.g. gene expression [5-13], signal transduction [14-17] and metabolic networks [18-25].

In this context, it is particularly worth mentioning the DREAM initiative (Dialogue for Reverse Engineering Assessments and Methods) [26], which targeted the problems of cellular network inference and quantitative model building in systems biology. DREAM tries to address two fundamental questions: (i) how can we assess how well we are describing the networks of interacting molecules that underlie biological systems? and (ii) how can we know how well we are predicting the outcome of previously unseen experiments from our models? Interestingly, one of the main conclusions of the DREAM3 event was that the vast majority of the teams' predictions were statistically equivalent to random guesses. Moreover, even for particular problem instances like gene regulation network inference, there was no one-size-fits-all algorithm [27].

The use of a performance profiling framework with the DREAM3 benchmark problems revealed that current inference methods are affected by different types of systematic prediction errors [6]. These authors conclude that reliable network inference from gene expression data remains an unsolved problem. Further, they highlight two major difficulties in the case of gene-network reverse engineering: limited data (which may leave the inference problem underdetermined), and the difficulty of distinguishing direct from indirect regulation. Prill et al [27] further explored the issue of intrinsic impediments to network inference, designating identifiability of certain network edges and systematic false positives as the main barriers. In this paper, we consider the widely used reaction kinetic formalism, where dynamic models of biological networks are described by a set of ordinary differential equations (see, e.g. [28-30] and the related literature). In particular, we consider the central question of the identifiability of such a network as understood in the systems and control area [31,32].

Identifiability analysis studies whether there is a theoretical chance of uniquely determining the parameters of a mathematical model assuming perfect noise-free measurements and error-free modeling [33-35]. One of the early approaches for identifiability testing of nonlinear models is based on the Taylor-series expansion of the system output using the fact that the Taylor coefficients are unique [36]. A similar but more general method uses the generating series or Volterra-series coefficients of the system which is the nonlinear generalization of the Laplace-transform method used for linear systems [37]. In [38] a similarity transformation approach is proposed that gives necessary and sufficient conditions on local and global identifiability through the checking of nonlinear controllability and observability conditions. The appearance of differential algebra methods in systems and control theory [39,40] opened the possibility for new types of identifiability tests that have gained significant popularity [41-43]. Further theoretical developments in the field include the identifiability conditions of rational function state space models [43], the possible effect of initial conditions on identifiability [44], and the application of Lie-algebras [45]. While identifiability is the property of a certain parameterized model, a related notion called distinguishability addresses the problem whether two or more parameterized models (with the same or with different structure) can produce the same output for any allowed input [46-48]. The literature about identifiability and distinguishability of biological and chemical system models is relatively wide: Compartmental systems (that form a special subclass of general mass-action networks) are studied in [38,49,50]. The authors treat general nonlinear CRNs in [51,52] and [53] where it is shown that for thermodynamically meaningful models, nonlinearity reduces the chance of indistinguishability compared to the linear case [54]. Geometric conditions for the indistinguishability of CRNs are given in [55] with a related comment in [56]. Computer algebra tools can be successfully used for the symbolic computations needed for identifiability and distinguishability testing of complex models [57-60].

The importance of identifiability has been recognized previously in systems biology, too [14,61-64]. However, and despite a number of works illustrating ways to test the structural and practical identifiability of models [65-67], a significant portion of modeling studies in systems biology continue to ignore this key property.

It has been known for long that chemical reaction networks with different structure and/or parametrization may produce the same dynamical models describing the time-evolution of species concentrations [28,55]. A related problem, namely the non-unique structure of Petri nets associated to reaction network dynamics, is studied in [68]. Additionally, the value of prior information in biological network inference was clearly shown in [69,70] by applying Bayesian network models. However, a constructive optimization-based approach for the study of dynamically equivalent (or similar) reaction networks is a recent development [71-74], which we further extend in this paper.

As a novelty, we present in this paper the definition and a computational method to find the so-called core reactions that are present in any dynamically equivalent reaction network if the set of complexes is given a priori. Moreover, a computationally improved method is introduced for the computation of dense realizations of CRNs together with a modified algorithm to check the uniqueness of a constrained reaction network structure. Structural non-uniqueness and the use of the proposed computational methods will be illustrated with the help of biological models known from the literature.

The structure of the paper is the following. The 'Methods' section introduces the notions of chemical reaction networks, structural identifiability and distinguishability of dynamical models. Moreover, it contains the procedures to obtain core reactions of a network and its sparse and dense representations, which rely on standard methods of linear programming (LP) and mixed integer linear programming (MILP) [75-78]. The analysis of four biological system models can be found in the 'Results and discussion' section, followed by the conclusions.

## Methods

The model class considered in this paper is of the following form

(1) $$\begin{array}{c} ẋ=f\left(x,u,\theta \right),\;\;\;x\left(\text{0}\right)=x_{0}\\ y=h\left(x,u,\theta \right), \end{array}$$

where *x *∈ ℝ*^n ^*is the state vector, *y *∈ ℝ*^m ^*is the output, *u *∈ ℝ*^k ^*is the input, and *θ *∈ ℝ*^d ^*denotes the parameter vector. We assume that the functions *f *and *h *are polynomial in the variables *x*, *u *and *θ*. Clearly, mass action type CRNs described in the following subsection (where *θ *is typically the set of reaction rate coefficients), and simple deterministic models of gene regulation such as the one in Example 4 belong to this model class.

### Basic notions and known results related to mass-action models

In this subsection, the basic definitions for the description of CRNs will be given together with the already published results on finding dynamically equivalent network realizations with certain prescribed properties.

#### Structural and dynamical description of mass-action networks

Following [79] and several other works, we will characterize CRNs with the following three sets.

1. $S=\left\{X_{1},\ldots ,X_{n}\right\}$ is the set of *species *or chemical substances.

2. $C=\left\{C_{1},\ldots ,C_{m}\right\}$ is the set of *complexes*. Formally, the complexes are represented as linear combinations of the species, i.e.

(2) $$C_{i}=\overset{n}{\underset{j=1}{\sum }}\alpha_{ij}X_{j},\;\;\;i=1,\ldots ,m,$$

where *α_ij _*are nonnegative integers and are called the *stoichiometric coefficients*.

3. $R=\{\left(C_{i},C_{j}\right)|C_{i},C_{j}\in C$, and *C_i _*is transformed to *C_j _*in the CRN} is the set of *reactions*. The relation $\left(C_{i},C_{j}\right)\in R$ will be denoted as *C_i _*→ *C_j_*. Moreover, a nonnegative weight, the *reaction rate coefficient *denoted by *k_ij _*is assigned to each reaction *C_i _*→ *C_j_*. Naturally, if the reaction *C_i _*→ *C_j _*is not present in the CRN then *k_ij _*= 0.

The above characterization naturally gives rise to the following graph structure (often called 'Feinberg-Horn-Jackson graph' or simply reaction graph) of a CRN [29]. The weighted directed graph *G *= (*V*, *E*) of a CRN consists of a finite nonempty set *V *of vertices and a finite set *E *of ordered pairs of distinct vertices called directed edges. The vertices correspond to the complexes, i.e. *V *= {*C*_1_, *C*_2_, ... *C_m_*}, while the directed edges represent the reactions, i.e. (*C_i_*, *C_j_*) ∈ *E *if complex *C_i _*is transformed to *C_j _*in the CRN. The positive reaction rate coefficients *k_ij _*are assigned as weights to the corresponding directed edges *C_i _*→ *C_j _*in the graph. (Edges corresponding to zero rate coefficients are not drawn in the reaction graph.) A set of complexes {*C*_1_, ..., *C_k_*} is called a *linkage class *of a CRN, if the complexes of the set are linked to each other in the reaction graph but not to any other complex. It is remarked that loops (i.e. directed edges that start and end at the same vertex) are not allowed in reaction graphs.

Assuming mass-action kinetics, the following dynamical description will be used to describe the time-evolution of species concentrations [29,79]:

(3) $$ẋ=Y\cdot A_{k}\cdot \psi \left(x\right),$$

where *x_i _*denotes the concentration of species *X_i_*. Let us denote the transpose and the (*i*, *j*)th element of an arbitrary matrix *W *by *W^T ^*and *W_i,j_*, respectively, where *i *is the row index and *j *is the column index. The *j*th column of *Y *contains the composition of complex *C_j_*, i.e. *Y_i,j _*= *α_ji_*. The structure and parameters of the reaction graph are stored in the column conservation matrix *A_k _*(also called the *Kirchhoff matrix *of the CRN) as follows

(4) $${\left[A_{k}\right]}_{i,j}=\left\{\begin{array}{cc} -\sum_{l=1,l\neq i}^{m}k_{il}, & \text{if}\;i=j\\ k_{ji}, & \text{if}\;i\neq j. \end{array}\right)$$

Finally, *ψ *: ℝ*^n ^*↦ℝ*^m ^*is a monomial-type vector mapping defined by

(5) $$\psi_{j}\left(x\right)=\overset{n}{\underset{i=1}{\prod }}x_{i}^{Y_{i,j}},\;\;\;j=1,\ldots ,m.$$

#### Dynamical equivalence of mass-action networks

As it is known even from the early literature [28], CRNs with different structures and/or parametrization can give rise to the same kinetic differential equations. Therefore, we will call two CRNs given by the matrix pairs $\left(Y^{\left(1\right)},A_{k}^{\left(1\right)}\right)$ and $\left(Y^{\left(2\right)},A_{k}^{\left(2\right)}\right)$*dynamically equivalent*, if

(6) $$Y^{\left(1\right)}A_{k}^{\left(1\right)}\psi^{\left(1\right)}\left(x\right)=Y^{\left(2\right)}A_{k}^{\left(2\right)}\psi^{\left(2\right)}\left(x\right)=f\left(x\right),\;\;\;\forall x\in {\bar{\mathbb{R}}}_{+}^{n},$$

where for $i=1,2,Y^{\left(i\right)}\in {\mathbb{R}}^{n\times m_{i}}$ have nonnegative integer entries, $A_{k}^{\left(i\right)}$ are valid Kirchhoff matrices, and

(7) $$\psi_{j}^{\left(i\right)}\left(x\right)=\overset{n}{\underset{k=1}{\prod }}x_{k}^{{\left[Y^{\left(i\right)}\right]}_{k,j}},\;i=1,2,\;j=1,\ldots ,m_{i}.$$

In this case, $\left(Y^{\left(i\right)}A_{k}^{\left(i\right)}\right)$ for *i *= 1, 2 are called *realization*s of a kinetic vector field *f *(see, e.g. [80] for more details). It is also appropriate to call $\left(Y^{\left(1\right)},A_{k}^{\left(1\right)}\right)$ a *realization *of $\left(Y^{\left(2\right)},A_{k}^{\left(2\right)}\right)$ and vice versa.

We will assume throughout the paper that the set of complexes (i.e. the stoichiometric matrix *Y*) is fixed and known before the computations. In this case, the condition (6) for dynamical equivalence can be written as

(8) $$Y\cdot A_{k}^{\left(1\right)}=Y\cdot A_{k}^{\left(2\right)}=:M,$$

where $A_{k}^{\left(1\right)}$ and $A_{k}^{\left(2\right)}$ are valid Kirchhoff matrices and *M *is the invariant matrix containing the coefficients of the monomials.

Among the dynamically equivalent realizations, it is important to recall the following characteristic ones described in [71,72]. A *sparse realization *contains the minimal number of reactions that is needed for the exact description of the corresponding dynamics (3). A *dense realization *contains the maximal number of reactions among dynamically equivalent realizations with a fixed complex set given by *Y*. While sparse realizations are generally structurally non-unique (as it will be illustrated for the constrained case, too, in Example 1), the structure of dense realizations with a given complex set is unique, and it contains every possible dynamically equivalent structure as a proper subgraph (i.e. a dense realization is a kind of super-structure) [71].

### Known computation approaches for finding preferred CRN realizations

Here we briefly summarize the already published results corresponding to the computation of preferred dynamically equivalent CRN realizations (more details of these methods can be found in the publications [71-73,81]). The computation of dense and sparse realizations can be traced back to mixed integer linear programming (MILP) where the decision variables are the non-diagonal elements of *A_k_*, the linear constraints encode the kinetic properties of the model, and the objective function contains integer variables for minimizing/maximizing the number of nonzero reaction rate coefficients [72]. It is remarked that the computation of sparse realizations is an NP-hard problem, where generally mixed integer linear programming cannot be avoided [82]. There exist certain conditions under which the problem can be solved in polynomial time [83] but these are often not fulfilled in the case of CRNs. Moreover, there are effective heuristics to address the problem [84], but convergence to one of the truly sparsest structures is not guaranteed. Luckily, the MILP-based computation of sparse CRN realizations can be parallelized effectively thus allowing a larger number of complexes to be treated. The computation of realizations having the minimal/maximal number of complexes or the reversibility property can also be solved in the MILP framework [71]. Moreover, it was shown in [73] that finding detailed balanced and complex balanced realizations of CRNs is a simple linear programming (LP) problem. Finally, weakly reversible dynamically equivalent CRN realizations can also be determined (if they exist) using MILP [85].

#### Constrained realizations of CRNs and testing their structural uniqueness

The following is a straightforward extension of the results published in [71]. To prove the uniqueness of a CRN structure given a set of simple constraints, we have to extend the notions of dense and sparse realizations. The constraint set denoted by K will be used for the exclusion of selected reactions from the CRN, i.e. it is of the form:

(9) $$K=\left\{{\left[A_{k}\right]}_{i_{1},j_{1}}=0,\;\ldots ,\;{\left[A_{k}\right]}_{i_{s},j_{s}}=0\right\},$$

where *s *is the number of individual constraints, and *i_k _*≠ *j_k _*for *k *= 1, ..., *s*. Now we can introduce the following definitions. A dynamically equivalent *constrained realization *of a CRN (*Y*, *A_k_*) is a reaction network $\left(Y,A_{k}^{\prime }\right)$ such that $Y\cdot A_{k}=Y\cdot A_{k}^{\prime }$ and the prescribed constraints K in the form of eq. (9) are fulfilled for $A_{k}^{\prime }$. A dynamically equivalent *constrained dense realization *of a CRN (*Y*, *A_k_*) is a constrained realization that contains the maximal number of nonzero elements in $A_{k}^{\prime }$. Similarly, the *constrained sparse realization *is a constrained realization with the minimal number of nonzeros in $A_{k}^{\prime }$. To characterize constrained dense/sparse realizations, the results of [71] can be adapted easily as follows.

**P1 **Given a CRN (*Y*, *A_k_*) and a constraint set K, the unweighted reaction graph of any constrained realization is the subgraph of the unweighted reaction graph of the constrained dense realization.

**P2 **If the sets of complexes and constraints are fixed, then for any CRN, the structure of the constrained dense realization is unique.

**P3 **The reaction graph structure of a CRN with given sets of complexes and constraints is unique if and only if the unweighted directed graphs of its constrained dense and sparse realizations are identical.

The proofs of **P1**, **P2 **and **P3 **follow similar (although not completely identical) lines that were published in [71], and they are given for convenience in the Appendix at the end of the paper.

### New concepts and computation results related to dynamically equivalent networks

This subsection contains new methodological contributions that extend the previously published results.

#### Making the computation of dense realizations more efficient

Computing dense realizations is treated originally also in a MILP-framework in [72]. However, using the structural uniqueness of such realizations given by **P1**, it is easy to give a polynomial-time algorithm based on a finite series of linear programming (LP) optimization steps. The idea of the improved algorithm is simple: the reaction *C_i _→ C_j _*belongs to the (constrained) dense realization if and only if there exists any dynamically equivalent (constrained) realization where [*A_k_*]*_j,i _>*0. The result directly follows from the fact that the unweighted reaction graphs of (constrained) dense realizations give a unique super-structure. This allows us to formulate a polynomial-time method based on pure LP to determine (constrained) dense realizations as follows.

The task of determining which reactions of a CRN belong to the dense realization can be effectively solved through the following problem set consisting of *m*(*m - *1) LP computation steps, where *m *is the number of complexes in the CRN.

(10) $$\begin{array}{c} \text{for each }p,q=1,\ldots ,m,\;p\neq q\;\text{do}:\\ \;\text{maximize }f_{pq}={\left[A_{k}\right]}_{p,q}\\ \;\text{subject to}:\\ \;\;Y\cdot A_{k}=M,\\ \;\;\overset{m}{\underset{i=1}{\sum }}{\left[A_{k}\right]}_{i,j}=0,\;j=1,\ldots ,m,\\ \;\;0\leq {\left[A_{k}\right]}_{i,j}\leq U_{ij},\;i,j=1,\ldots ,m,\;i\neq j,\\ \;\;{\left[A_{k}\right]}_{i,i}\leq 0,\;i=1,\ldots ,m, \end{array}$$

where the decision variables are the off-diagonal entries of *A_k_*, and *U_ij _*are appropriately large positive upper bounds for [*A_k_*]*_i,j _*to exclude the possibility of unbounded feasible solutions. The reaction *C_q _*→ *C_p _*is in the dense realization if and only if the maximal objective function value for *f_pq _*in (10) is positive. Let us denote the solution of (10) corresponding to (*p*, *q*), *p *≠ *q *by ${\bar{A}}_{k}^{pq}$. Since the linear equality and inequality constraints in (10) are trivially convex, we will use the average of the obtained solutions ${\bar{A}}_{k}^{pq}$ as a lower bound to compute a possible dense realization in the final optimization step. For this, we define

(11) $$\varepsilon_{ij}={\left[\begin{array}{cc} \frac{1}{m\left(m-1\right)} & \overset{m}{\underset{\begin{array}{c} p,q=1\\ p\neq q \end{array}}{\sum }}{Ā}_{k}^{pq} \end{array}\right]}_{i,j}\;,i\neq j.$$

By construction, *ε_ij _*≥ 0 ∀*i *≠ *j*, and *ε_ij _>*0 if and only if the reaction $C_{j}\to C_{i}$ is in the dense realization. Then the actual dense realization can be determined by solving the following LP feasibility problem for *A_k _*(with arbitrary linear objective function):

(12) $$\begin{array}{c} Y\cdot A_{k}=M,\\ \overset{m}{\underset{i=1}{\sum }}{\left[A_{k}\right]}_{i,j}=0,\;\;j=1,\ldots ,m,\\ \varepsilon_{ij}\leq {\left[A_{k}\right]}_{i,j}\leq U_{ij},\;\;i,j=1,\ldots ,m,\;\;i\neq j,\\ {\left[A_{k}\right]}_{i,i}\leq 0,\;\;i=1,\ldots ,m. \end{array}$$

It is important to remark that the definition of *ε_ij _*in the form of (11) guarantees the solvability of (12). Naturally, the above described method can also be used for determining constrained dense realizations by adding constraints of the form (9) to the LP problems (10) and (12).

Using the notion and described properties of constrained realizations, we are now able to test the structural uniqueness of given CRNs. To accomplish this, only the (constrained) dense and sparse realizations have to be computed and compared (see **P3**). This method will be illustrated in Example 2.

#### Definition and computation of core and non-core reactions

We will call a reaction a *core reaction*, if it is present in any dynamically equivalent realization of a CRN with a given complex set (and possibly an additional constraint set). Other reactions, the rate coefficient of which can be zero in certain realizations, are called *non-core reactions*. It clearly follows from the definition, but is remarked separately that the set of core reactions is generally not identical to the set of reactions of a sparse realization. The identification of core reactions of a CRN has not been published yet, therefore we give the outline of the corresponding computation method. Firstly, a dense realization of the network has to be computed to get all the mathematically possible reactions. Then, for each reaction *C_p _*→ *C_q _*in the dense realization, the feasibility of the following constraint set has to be checked:

(13) $$Y\cdot A_{k}=M$$

(14) $$\overset{m}{\underset{i=1}{\sum }}{\left[A_{k}\right]}_{i,j}=0,\;\;\;j=1,\ldots ,m$$

(15) $${\left[A_{k}\right]}_{i,j}\geq 0,\;\;\;i,j=1,\ldots ,m,\;\;\;i\neq j,\;\;\left(i,j\right)\neq \left(q,p\right)$$

(16) $${\left[A_{k}\right]}_{i,i}\leq 0,\;\;\;i=1,\ldots ,m$$

(17) $${\left[A_{k}\right]}_{q,p}=0,$$

where the matrix *A_k _*contains the decision variables, and the known matrices are *Y *and *M*. It is well-known that this task is equivalent to an LP problem where the objective function is an arbitrary linear function of the elements of *A_k _*[76]. Then, reaction *C_p _*→ *C_q _*is a core-reaction if and only if the set defined by (13)-(17) is empty (i.e. the corresponding LP problem is infeasible), because in this case there is no dynamically equivalent CRN realization where *C_p _*→ *C_q _*is not present. We remark here that the presented procedures for determining constrained dense realizations and computing core reactions are parallel in their original forms since the individual LP steps are independent of each other. Therefore the proposed methods can be very effectively implemented in a grid or multi-core hardware environment [86].

### Basic concepts on structural identifiability and distinguishability

Let us recall eq. (1). Shortly speaking, global structural identifiability means that

(18) $$ŷ\left(t|\theta^{\prime }\right)\equiv ŷ\left(t|\theta^{\prime \prime }\right)\Rightarrow \theta^{\prime }=\theta^{\prime \prime },$$

where

(19) ŷ(t|θ)=h(x(t,θ),u(t),θ),

and *x*(*t*, *θ*) denotes the solution of (1) with parameter vector *θ*. According to (18), a structurally non-identifiable model can produce exactly the same observed output with different parametrization. This is clearly a fundamental obstacle of determining the true model parameters from measurements even if the selected model structure is considered to be correct.

Let us denote two parameterized models with possibly different structure by $M_{1}\left(\theta_{1}\right)$ and $M_{2}\left(\theta_{2}\right)$, respectively, where *θ_i _*denote the parameter vector. Then $M_{1}$ is called *distinguishable *from $M_{2}$ if for any *θ*_1 _(possibly except for a finite number of values) there is no *θ*_2 _such that the input-output behaviour of $M_{1}$ and $M_{2}$ is the same [47]. Clearly, if $M_{1}$ and $M_{2}$ are indistinguishable and both model structures are feasible in a certain application, then there is no way to decide from input-output measurements to which one corresponds to the true model that generated the data.

In the case of CRNs, we will assume that all species concentrations are measured (i.e. *y *= *x*), the input is zero (i.e. we study autonomous systems), and that the set of possible chemical complexes is given. Generally, the model parameter vector *θ *is the set of reaction rate coefficients which are the off-diagonal elements of *A_k_*. Clearly, if a CRN has several different dynamically equivalent realizations, then these realizations are not distinguishable without additional constraints, and the model cannot be identifiable if all the rate coefficients are to be determined [55]. This situation can be improved by using prior knowledge in the form of adding further constraints on the model parameters such as the simple ones given by eq. (9). This way, the number of parameters to be estimated can be reduced and/or their feasibility region can be shrinked. It is important to note that although the structural uniqueness of a CRN definitely reduces the degree of non-identifiability (since zero and non-zero parameters are separated), it does not necessarily imply structural identifiability [53], and this latter property has to be checked by further numerical methods [31,32].

## Results and discussion

In this section, the application of the previously mentioned methods for finding different dynamically equivalent structures will be illustrated using biological models taken from the literature. The detailed numerical data corresponding to Examples 1-3 are contained in a standard spreadsheet form with brief explanations in Additional file 1: CRN_data.xls.

### Example 1: a positive feedback motif

**Figure 1:**
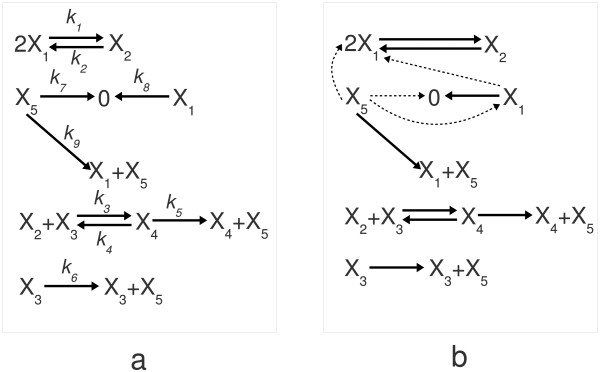
**Positive feedback motif: original reaction graph and dense realization structure**. (a) This subfigure shows the reaction graph of a gene regulation network model with positive feedback described originally in [87] and used in Example 1. (b) This subfigure shows all the mathematically possible reactions that can result in the same dynamical behaviour as the original biologically meaningful network shown in Figure 1. The core-reactions in the dense realization are shown with solid arrows, while the non-core reactions are indicated by dashed arrows.

The first example is a positive feedback motif shown in Figure 1a and taken from [87] containing 5 species, 11 complexes and 9 reactions. This basic motif is also discussed in [88]. The network contains a gene that promotes its own transcription and translation after dimerization. In the model, *X*_1 _and *X*_2 _denote the concentrations of protein monomers and dimers, respectively. *X*_3 _and *X*_4 _are the concentrations of unoccupied and occupied promoters, respectively, and *X*_5 _corresponds to the mRNA. The degradation of dimers is ignored. The roles of the reaction rate coefficients are the following: *k*_1 _and *k*_2 _are the dimerization and re-dimerization rates, respectively. *k*_3 _and *k*_4 _are the binding and dissociation rates of the dimer to the promoter, while *k*_5 _and *k*_6 _denote the activated and basal transcription rates, respectively. *k*_7 _is the degradation rate of the mRNA, *k*_8 _is the degradation rate of the monomer, and *k*_9 _denotes the translation rate. The time-evolution of the species-concentrations is described by the following ODEs:

(20) $${ẋ}_{1}=-2k_{1}x_{1}^{2}+2k_{2}x_{2}+k_{9}x_{5}-k_{8}x_{1}$$

(21) $${ẋ}_{2}=k_{1}x_{1}^{2}-k_{2}x_{2}-k_{3}x_{2}x_{3}+k_{4}x_{4}$$

(22) $${ẋ}_{3}=-k_{3}x_{2}x_{3}+k_{4}x_{4}$$

(23) $${ẋ}_{4}=k_{3}x_{2}x_{3}-k_{4}x_{4}$$

(24) $${ẋ}_{5}=k_{5}x_{4}+k_{6}x_{3}-k_{7}x_{5}.$$

Our starting point is that we have a dynamic model of the process in the standard polynomial form of (20)-(24), the parameters of which are known from the results of identification and/or from literature. As we will see below, without well-defined constraints on the possible set of complexes and reactions, exactly the same dynamics can be realized in principle by a wide range of mechanisms.

The matrices characterizing the stoichiometry and graph structure of the system are the following (indicating only the nonzero non-diagonal elements of *A_k_*):

(25) $$Y=\left[\begin{array}{ccccc} 2 & 0 & 0 & 0 & 0\\ 1 & 0 & 0 & 0 & 1\\ 1 & 0 & 0 & 0 & 0\\ 0 & 1 & 1 & 0 & 0\\ 0 & 1 & 0 & 0 & 0\\ 0 & 0 & 1 & 0 & 1\\ 0 & 0 & 1 & 0 & 0\\ 0 & 0 & 0 & 1 & 1\\ 0 & 0 & 0 & 1 & 0\\ 0 & 0 & 0 & 0 & 1\\ 0 & 0 & 0 & 0 & 0 \end{array}\right]$$

(26) $$\begin{array}{c} {\left[A_{k}\right]}_{2,1}=k_{1},\;{\left[A_{k}\right]}_{1,2}=k_{2},\;{\left[A_{k}\right]}_{4,3}=k_{3},\\ {\left[A_{k}\right]}_{3,4}=k_{4},\;{\left[A_{k}\right]}_{5,4}=k_{5},\;{\left[A_{k}\right]}_{7,6}=k_{6},\\ {\left[A_{k}\right]}_{9,8}=k_{7},\;{\left[A_{k}\right]}_{9,10}=k_{8},\;{\left[A_{k}\right]}_{11,8}=k_{9}. \end{array}$$

**Figure 2:**
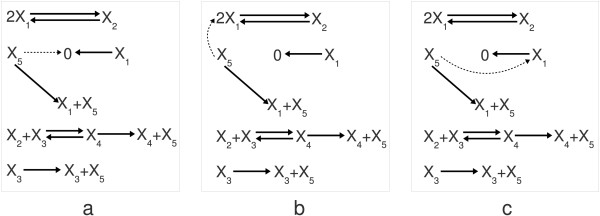
**Sparse realization structures for the positive feedback motif**. Three different dynamically equivalent structures can be given for the positive feedback motif with the minimal number of reactions. The core and non-core reactions are indicated in the same way as in Figure 1.b.

We used the following parameter values that were taken from the Appendix of [87].

(27) $$\begin{array}{c} k_{1}=k_{2}=k_{3}=k_{4}=10^{7},\;k_{5}=1.7,\\ k_{6}=0.025,\;k_{7}=0.1,\;k_{8}=0.05,\;k_{9}=0.5, \end{array}$$

where the units of measure are [M^-1^] for *k*_1_, ..., *k*_4_, and [min^-1^] for *k*_5_, ..., *k*_9_. The dynamically equivalent dense realization of the network is shown in Figure 1b, where the 8 core and 4 non-core reactions are indicated separately. The three different sparse structures are shown in the subplots of Figure 2. The first subplot is identical to the original structure shown in Figure 1a. This means that the mechanism cannot be described exactly with less than 9 reactions. It turns out from the second and third subplots that (at least mathematically), the degradation of mRNA is dynamically not a necessary element of the model. However, the biological plausibility of the mathematically possible structures and reactions always has to be carefully examined.

**Figure 3:**
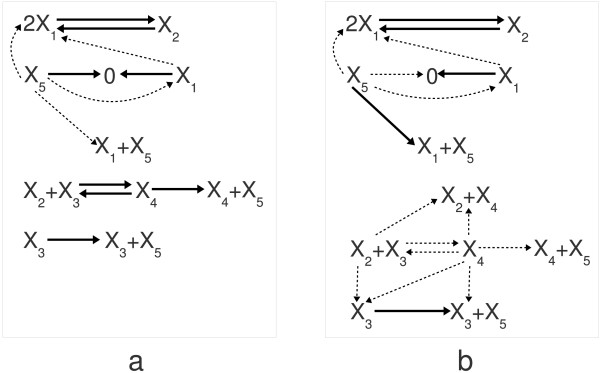
**The effect of modifying the complex set and the parameters**. (a) The core and non-core reactions of the dense realization of the positive feedback motif are shown in this subfigure with a randomly selected parametrization that is different from the one given in [87]. (b) The core and non-core reactions of the dense realization of the positive feedback motif can be seen in this subfigure when an additional complex *X*_2 _+ *X*_4 _is involved into the model.

As it is expected, the possible structures of sparse/dense realizations and the corresponding core and non-core reactions can change with the modification of parameter values. This is illustrated in Figure 3a, where the following randomly generated parameter values were used:

(28) $$\begin{array}{c} k_{1}=18.9,\;k_{2}=7.1,\;k_{3}=15.4,\\ k_{4}=12.7,\;k_{5}=10.6,\;k_{6}=3.5,\\ k_{7}=11.3,\;k_{8}=9.1,\;k_{9}=4.0. \end{array}$$

It is visible that the structure of the dense realization is the same as in Figure 1b but the core reactions are different from the ones shown there. Here the degradation of mRNA is described by a core reaction but interestingly, the reaction corresponding to translation is not a core one. Naturally, this implies that the possible sparse realization structures with the second parametrization are different from the ones shown in Figure 2. Note that here the only goal was to illustrate the possible change of core and non-core reactions, and therefore the biological relevance of the parameter values in eq. (28) is not assumed in this case.

In the next step, let us assume that another complex, namely *X*_2 _+ *X*_4 _is allowed in the model (again not necessarily assuming biological meaningfulness in this particular case). With the addition of this new complex, the stoichiometric matrix of the system can be written as

(29) $$Y^{\prime }={\left[\begin{array}{ccccc} 2 & 0 & 0 & 0 & 0\\ 1 & 0 & 0 & 0 & 1\\ 1 & 0 & 0 & 0 & 0\\ 0 & 1 & 1 & 0 & 0\\ 0 & 1 & 0 & 0 & 0\\ 0 & 0 & 1 & 0 & 1\\ 0 & 0 & 1 & 0 & 0\\ 0 & 0 & 0 & 1 & 1\\ 0 & 0 & 0 & 1 & 0\\ 0 & 0 & 0 & 0 & 1\\ 0 & 0 & 0 & 0 & 0\\ 0 & 1 & 0 & 1 & 0 \end{array}\right]}^{T}.$$

The dense CRN realization of the dynamics (20)-(24) with the updated *Y*' matrix given in eq. (29) using the original parameters described in (27) is shown in Figure 3b, where the core and non-core reactions are again indicated. It is apparent that now there are only 5 core reactions, and none of the remaining 12 reactions are essential to represent the dynamics (20)-(24). This means that the introduction of a new complex increased the flexibility of the network (i.e. mathematically, the majority of the reactions can be substituted by other ones and the network still maintains its original dynamics). Of course, not any combination of the non-core reactions can be omitted from the network, because the sparse realizations show that at least 9 reactions are needed to keep dynamical equivalence. It can be computed easily that the theoretical maximum number of sparse realizations with different structures is $\left(\begin{array}{c} 12\\ 17-9 \end{array}\right)=495$. However, as the numerical experiments show, majority of these structures do not give a practically feasible dynamically equivalent realization.

The above results clearly show that certain mechanisms may remain undetectable (or they are falsely detected) even if we have complete species concentration measurements and full information about possible complex formation, that are not very realistic assumptions. Moreover, the sparsest dynamically equivalent structure of mass-action models is not unique, therefore sparsity enforcing approaches for determining 'true' reaction structures are not enough in themselves without the necessary amount of prior information given in the form of additional constraints. The practical situation is most often even worse than that, since the measurements are typically incomplete, noisy and sometimes dynamically not rich enough, that may introduce further obstacles to the structure/parameter estimation process [66,89].

### Example 2: a biochemical switch in yeast cells

**Figure 4:**
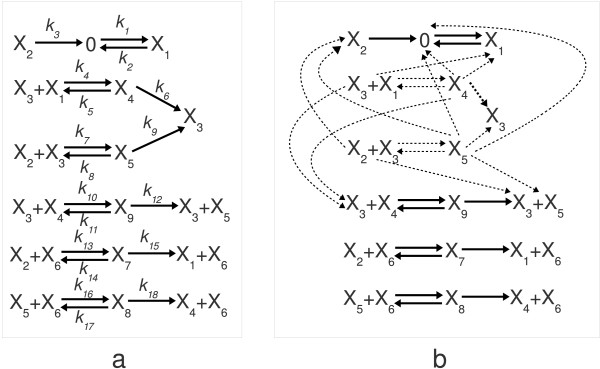
**Model of a biochemical switch in yeast cells**. (a) The subfigure shows the original structure of a CRN describing a biochemical switch published in [90]. The numbering of species and rate coefficients is identical to the description in the original paper. (b) The dense realization of the network is depicted in this subfigure and contains 28 reactions, out of which only 12 belong to the set of core reactions.

The following example is taken from [90] and it describes a 'switching device' in yeast cycle regulation. The detailed system description can be found in [90] and in the accompanying supporting information document. The order of state variables, corresponding to concentrations, is the same as in the original article, and is shown below:

*x*_1_: [Sic1], *x*_2_: [Sic1P], *x*_3_: [Clb], *x*_4_: [Clb·Sic1], *x*_5_: [Clb·Sic1P], *x*_6_: [Cdc14], *x*_7_: [Sic1P·Cdc14], *x*_8_: [Clb·Sic1P·Cdc14], *x*_9_: [Clb·Sic1·Clb]. The original structure with 18 reactions is shown in Figure 4a. The *Y *matrix of the network is given by

(30) $$Y={\left[\begin{array}{ccccccccc} 0 & 1 & 0 & 0 & 0 & 0 & 0 & 0 & 0\\ 0 & 0 & 0 & 0 & 0 & 0 & 0 & 0 & 0\\ 1 & 0 & 0 & 0 & 0 & 0 & 0 & 0 & 0\\ 1 & 0 & 1 & 0 & 0 & 0 & 0 & 0 & 0\\ 0 & 0 & 0 & 1 & 0 & 0 & 0 & 0 & 0\\ 0 & 0 & 1 & 0 & 0 & 0 & 0 & 0 & 0\\ 0 & 1 & 1 & 0 & 0 & 0 & 0 & 0 & 0\\ 0 & 0 & 0 & 0 & 1 & 0 & 0 & 0 & 0\\ 0 & 0 & 1 & 1 & 0 & 0 & 0 & 0 & 0\\ 0 & 0 & 0 & 0 & 0 & 0 & 0 & 0 & 1\\ 0 & 0 & 1 & 0 & 1 & 0 & 0 & 0 & 0\\ 0 & 1 & 0 & 0 & 0 & 1 & 0 & 0 & 0\\ 0 & 0 & 0 & 0 & 0 & 0 & 1 & 0 & 0\\ 1 & 0 & 0 & 0 & 0 & 1 & 0 & 0 & 0\\ 0 & 0 & 0 & 0 & 1 & 1 & 0 & 0 & 0\\ 0 & 0 & 0 & 0 & 0 & 0 & 0 & 1 & 0\\ 0 & 0 & 0 & 1 & 0 & 1 & 0 & 0 & 0 \end{array}\right]}^{T}.$$

The non-zero off-diagonal elements of *A_k _*are (the diagonal ones can be computed using the column conservation property):

(31) $$\begin{array}{c} {\left[A_{k}\right]}_{2,1}=k_{3},\;{\left[A_{k}\right]}_{2,3}=k_{2},\;{\left[A_{k}\right]}_{3,2}=k_{1},\\ {\left[A_{k}\right]}_{4,5}=k_{5},\;{\left[A_{k}\right]}_{5,4}=k_{4},\;{\left[A_{k}\right]}_{6,5}=k_{6},\\ {\left[A_{k}\right]}_{6,8}=k_{9},\;{\left[A_{k}\right]}_{7,8}=k_{8},\;{\left[A_{k}\right]}_{8,7}=k_{7},\\ {\left[A_{k}\right]}_{9,10}=k_{11},\;{\left[A_{k}\right]}_{10,9}=k_{10},\;{\left[A_{k}\right]}_{11,10}=k_{12},\\ {\left[A_{k}\right]}_{12,13}=k_{14},\;{\left[A_{k}\right]}_{13,12}=k_{13},\;{\left[A_{k}\right]}_{14,13}=k_{15},\\ {\left[A_{k}\right]}_{15,16}=k_{17},\;{\left[A_{k}\right]}_{16,15}=k_{16},\;{\left[A_{k}\right]}_{17,16}=k_{18}. \end{array}$$

Since there are no parameter values published in [90], we used the following randomly selected rate coefficients:(32)

The structure of the dense realization indicating the 12 core and 16 non-core reactions can be seen in Figure 4b.

It can be shown using the computational methods described in the 'Methods' section that the only possible sparse realization structure is identical to that of the original network. Therefore in this special case, there is only one possible reaction structure containing the minimal number of reactions. A straightforward approach to ensure the structural uniqueness of the whole network is to exclude all reactions that are not meaningful from the examined application's point of view or that are contradictory to modeling assumptions. For the current example, the removal of an unexpectedly low number of reactions is enough to obtain a unique structure. It can be shown by computing the corresponding constrained dense and sparse realizations, that excluding the reactions *X*_5 _→ *X*_3 _+ *X*_5_, *X*_4 _→ *X*_3 _+ *X*_4_, *X*_2 _+ *X*_3 _→ *X*_3 _+ *X*_5_, and *X*_3 _+ *X*_1 _→ *X*_3 _+ *X*_4 _is enough to make the reaction structure unique that is identical to the original structure shown in Figure 4. In other words, the exclusion of 4 well-selected reactions leads to the removal of an additional 6 reactions leaving only 18.

### Example 3: a repressilator structure with 5 nodes and auto-activation

**Figure 5:**
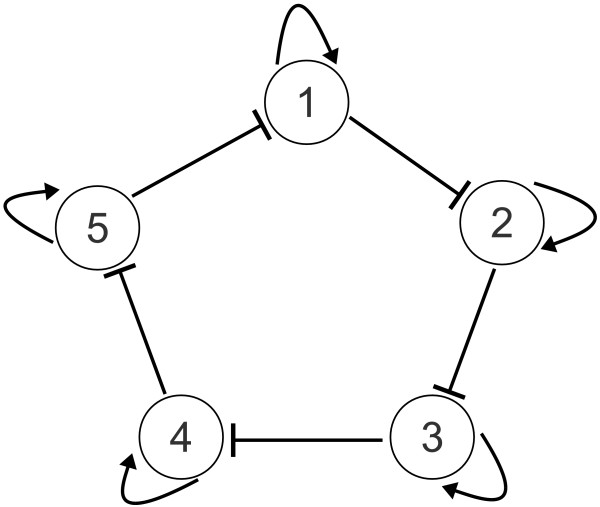
**A standard repressilator structure**. A repressilator structure with 5 nodes and auto-activation is shown in the figure. The mass-action type CRN model of this structure contains 51 distinct complexes and 55 reactions.

Consider the repressilator model shown in Figure 5 with 5 nodes where also auto-activation is assumed. Similarly to [91], we make the following assumptions: cooperative regulator binding, genes are present in constant amounts, transcription and translation are modeled by single-step kinetics, and finally, proteins are degraded by first order reactions. We note that complex dynamic phenomena such as multiple steady states or oscillations have been shown with a wide range of parameters in similar systems, especially in the case when the number of genes is odd [91]. We also assume that there is some protein production (leakage) when both the activator and the repressor are bound to the genes (although this assumption does not affect the main results of the forthcoming analysis). It is clearly shown in [92] that kinetic models with simple mass-action kinetics very effectively describe complex dynamics in genetic regulatory networks, therefore we follow the same modeling methodology. Using the assumptions listed above, the CRN describing the system is the following:

(33) $$G_{i}+P_{i}\overset{k_{i,1}}{\underset{k_{i,2}}{⇄}}G_{i}^{A}\;\left(\text{auto-activation}\;1\right)$$

(34) $$G_{i}^{A}\overset{k_{i,3}}{\to }G_{i}^{A}+P_{i}\;\left(\text{protein}\;\text{production}\;1\right)$$

(35) $$G_{i}+P_{j}\overset{k_{i,4}}{\underset{k_{i,5}}{⇄}}G_{i}^{R}\;\left(\text{repression}\;1\right)$$

(36) $$G_{i}^{R}+P_{i}\overset{k_{i,6}}{\underset{k_{i,7}}{⇄}}G_{i}^{AR}\;\left(\text{auto-activation}\;2\right)$$

(37) $$G_{i}^{A}+P_{j}\overset{k_{i,8}}{\underset{k_{i,9}}{⇄}}G_{i}^{AR}\;\left(\text{repression}\;2\right)$$

(38) $$G_{i}^{AR}\overset{k_{i,10}}{\to }G_{i}^{AR}+P_{i}\;\left(\text{protein production}\;2\right)$$

(39) $$P_{i}\overset{k_{i,11}}{\to }0\;\left(\text{protein degradation}\right)$$

for the index pairs (*i*, *j*) ∈ {(1, 5), (2, 1), (3, 2), (4, 3), (5, 4)}. In eqs. (33)-(39), *G_i _*and *P_i _*represent the *i*th gene and protein, respectively. For the genes, superscripts *A *and *R *refer to activated and repressed states, respectively. Let us denote with *r_i,k _*the reaction with rate coefficient *k_i,k _*in eqs. (33)-(39).

Two cases with different sets of randomly selected rate coefficients were studied, and the structures of the obtained results were the same. The numerical details can be found on the 3rd sheet of Additional File 1: CRN_data.xls. The total number of reactions for the repressilator model is 55 that is equal to the number of reactions in the sparse realization. The dense realization contains 70 reactions which means that there are a maximum of 15 more mathematically possible reactions while maintaining exactly the same dynamics as the original biological model. These additional reactions are the following:

(40) $$\begin{array}{c} G_{i}^{AR}\to G_{i}^{R},\;P_{i}+G_{i}^{R}\to G_{i}^{R},\\ P_{i}+G_{i}^{R}\to P_{i}+G_{i}^{AR},\;\text{for}\;i=1,\;\ldots \;,5. \end{array}$$

The number of core reactions in the model are 45. The set of non-core reactions (that, in principle can be substituted by other reactions) is given by

(41) $$G_{i}^{AR}⇆G_{i}^{R}+P_{i},\;i=1,\;\ldots \;,5.$$

In particular, it is easy to show (see also Additional File 1: CRN_data.xls) that reactions $G_{i}^{AR}\to G_{i}^{R}+P_{i}$ and $G_{i}^{AR}\to G_{i}^{R}$ are always indistinguishable. Similarly, the reaction $G_{i}^{R}+P_{i}\to G_{i}^{AR}$ can be substituted with the combination of reactions $G_{i}^{R}+P_{i}\to G_{i}^{R}$ and $G_{i}^{R}+P_{i}\to G_{i}^{AR}+P_{i}$. It can be seen from these results that in order to have a model with unique structure, it is very important to a priori exclude all reactions that are not meaningful for the particular application.

### Example 4: sparse linear gene regulation network models

For structural identification, gene regulation networks are often modeled as linear time-invariant systems [84,93] of the form

(42) ẋ=Ax+Bu,

where *A *∈ ℝ*^n×n ^*contains the connectivity information of the network. *A_i,j _>*0 indicates activation from node *j *to node *i*, while *A_i,j _<*0 means repression, diagonal elements of *A *represent auto-activation or auto-repression depending on their sign. *x *∈ ℝ*^n ^*is the fully or partially measurable state of the system describing the time evolution of concentrations, and the input part *Bu *represents experimental perturbation (e.g. activation) of the genes. It is also a common assumption that the network is 'sparse' which means that there are only a limited number of activation or repression links between the nodes (i.e. the matrix *A *is 'sparse', too). But assuming sparsity can be a serious obstacle to identifiability as it will be shown.

**Figure 6:**
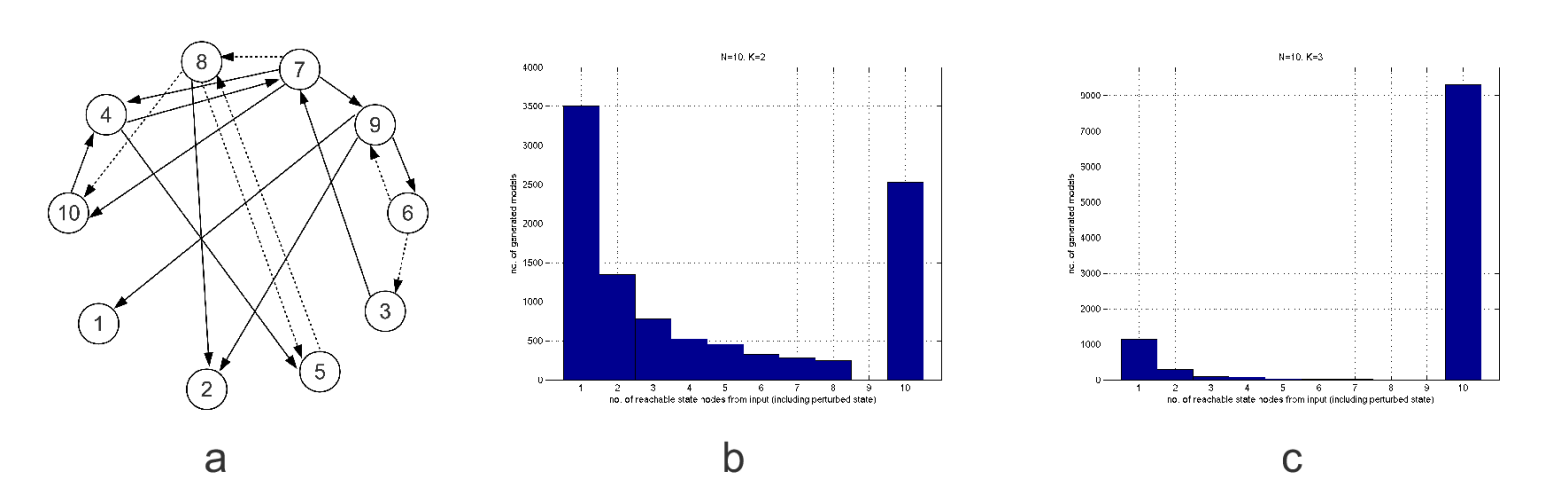
**A sparse gene regulation network and their structural identifiability properties**. (a) This subfigure is the reproduction of one of the sparse gene regulation networks used for structural identification in [93]. The network has 11 activation (solid edges), 6 suppression (dashed edges), and 3 autoregulation links (at nodes 1, 3 and 6) with undefined sign. (b) The subfigure shows that the vast majority of the randomly generated models in the case when *N *= 2 and *K *= 2 are structurally unidentifiable, because not all nodes of the network are reachable from the perturbed one. (c) As the network becomes less sparse (*N *= 10, *K *= 3), the structural identifiability properties are quickly improving. In this case, more than 80% of the randomly generated models are structurally identifiable.

First, consider the 'true' genetic network structure that was simulated and inferred in [93] and that is redrawn in Figure 6a. From the figure, we can reconstruct the structure of the corresponding *A *matrix as follows (the exact parameter values are not described in the paper, but the investigated structural properties do not depend on the individual parameter values)

(43) $$\left[\begin{array}{cccccccccc} {}* & 0 & 0 & 0 & 0 & 0 & 0 & 0 & + & 0\\ 0 & 0 & 0 & 0 & 0 & 0 & 0 & + & + & 0\\ 0 & 0 & * & 0 & 0 & - & 0 & 0 & 0 & 0\\ 0 & 0 & 0 & 0 & 0 & 0 & + & 0 & 0 & +\\ 0 & 0 & 0 & + & 0 & 0 & 0 & - & 0 & 0\\ 0 & 0 & 0 & 0 & 0 & * & 0 & 0 & + & 0\\ 0 & 0 & + & + & 0 & 0 & 0 & 0 & 0 & 0\\ 0 & 0 & 0 & 0 & - & 0 & - & 0 & 0 & 0\\ 0 & 0 & 0 & 0 & 0 & - & + & 0 & 0 & 0\\ 0 & 0 & 0 & 0 & 0 & 0 & + & - & 0 & 0 \end{array}\right],$$

where '+', '-' and '*' represent positive, negative and nonzero (but otherwise undefined) parameter values, respectively. If there are no prior assumptions about the structure of the interconnection matrix or about the relations between certain parameters, we can easily test the structural non-identifiability of the model by checking whether all nodes are reachable from the perturbed node on a directed path in the interconnection graph or not [33]. The reachability of nodes can be tested by several methods, e.g. a depth- or breadth-first-search (DFS or BFS) of the corresponding directed graphs that are fast polynomial-time algorithms [94]. To give a very simple example, it is clear from Figure 6a, that if nodes 1 or 2 were excited by an input signal, then the connections between the other nodes (3-10) would be undetectable by any method, however sophisticated it is. To examine whether situations like this one are common, we generated 10000 random state space models using the same method, and assuming zero initial conditions as in [93]. The connectivity of the corresponding directed graphs was tested using DFS. For 10 nodes and 2 nonzero elements in each row of *A *(i.e. *N *= 10, *K *= 2), we obtained that 73.38% of the generated models are structurally non-identifiable. The histogram showing the number of reachable states is shown in Figure 6b. The situation is dramatically improving if K is increased to 3 as shown in Figure 6c. In this case, around 17% of the models are structurally non-identifiable. When we have 20 nodes and 5 nonzero elements in each row of A (the second case investigated in [93]), then only 1.6% of the generated models are structurally non-identifiable. The results show that 'sparsity' has a clearly negative effect on structural identifiability because of limited information transmission between nodes. And finally, we did not speak at all about practical identifiability which is known to be a challenging issue even if the required structural properties are fulfilled [66].

### Relation between high level networks and CRN structure

As shown in Example 3, the various possible dynamically equivalent CRN structures do not correspond to a different GRN structure, if all species concentration measurements are available and the mapping described in [92] is used for transforming the models into each other. Hence, exact matching of the dynamics of different GRN structures may generally be a too severe restriction. To extend this line of research, the relaxation of dynamical equivalence to 'close dynamical similarity' seems to be more meaningful but the corresponding definitions and computational methods are much more complex than in the case of dynamical equivalence. One promising recent approach to assess dynamical similarity of CRNs (that also adds more degrees of freedom to the computations) is the concept of 'linear conjugacy' [74]. However, it might happen that dynamically completely equivalent GRN structures will be shown in the future.

## Conclusions

It has been shown in this paper using illustrative examples that biological network structures modeled by CRNs often cannot be uniquely determined even if the structure of the corresponding mathematical model is assumed to be known and all dynamical variables are measurable. The structural uniqueness and identifiability of the models often require additional constraints.

The main new contributions of the paper are the following. Firstly, core reactions present in any dynamically equivalent CRN realizations with a given complex set have been defined and a simple procedure with polynomial time-complexity has been given for determining them. Clearly, the core reactions are mandatory elements of every dynamically equivalent CRN realization assuming a fixed complex set. Secondly, a polynomial-time method based on linear programming for computing dense realizations has been outlined that is more scalable and therefore presents a clear improvement over the previously used MILP-based method. As an additional minor extension of previous results, constrained realizations of CRNs have been defined, and a computational method has been proposed to check the uniqueness of constrained realizations.

The presented concepts and algorithms were illustrated on previously published models describing biological processes. It was shown that the set of core reactions may change with the modification of the complex set. The examples also show that the frequently applied sparsity assumption alone is not enough for structural uniqueness of CRNs. Moreover, in the case of simple linear genetic network models, too sparse structures can degrade identifiability properties. The results further support the fact that as much prior information as possible should be incorporated in structural and parametric inference problems.

## Competing interests

The authors declare that they have no competing interests.

## Authors' contributions

All authors contributed to the conception and design of the work. JRB and GS selected and evaluated the examples. GS performed the numerical computations. All authors contributed to the writing of the manuscript. All authors read and approved the final manuscript.

## A Appendix

*Proof of ***P1**. Let us denote the *i*th column of any matrix *W *by *W*_·,*i*_. The proof is based on the following well known fact of linear algebra. Consider an inhomogeneous set of linear equations:

(44) Ax=b

If *x *= *p *is any particular solution of (44) then the entire solution set for (44) can be characterized as

(45) {p+v|visanysolutionofAx=0}

The matrix equation *Y · A_k _*= *M *(see eqs. (3) and (8)) obviously defines *m *sets of linear equations of the form

(46) $$Y\cdot {\left[A_{k}\right]}_{\cdot ,i}=M_{\cdot ,i},\;i=1,\ldots ,m$$

Let us choose any *i *indexing the sets of equations in (46). For simplicity, let *p *= [*A_k_*]_·,*i*_, *b *= *M*_·,*i*_. Let us assume that there are *z *elements of the constraint set (9) where *j_k _*= *i *for *k *= 1, ..., *s*. (If *z *is 0, then we get the earlier result proved in [71].) These constraints can be expressed by further linear equations of the form:

(47) $${\left[A_{k}\right]}_{h,i}=0,\;h=1,\;\ldots ,\;z$$

The equation sets (46) and (47) can be written into a single set of equations as

(48) $$Ȳ\cdot p=\bar{b}$$

where $Ȳ\in {\mathbb{R}}^{\left(n+z\right)\times m}$ and $\bar{b}\in {\mathbb{R}}^{n+z}$. Let us assume now that *p *is a dense solution for (48), i.e. it contains the maximal possible number of nonzero elements. If *p *has no zero elements, then the result to be proved is trivially satisfied. Therefore, without the loss of generality we can assume that the first *l < m *elements of p are nonzero, while the rest are zero, i.e. *p_j _*≠ 0 for *j *= 1, ..., *l*, and *p_j _*= 0 for *j *= *l *+ 1, ..., *m*. This can always be achieved by the appropriate reordering of the elements of *p*. Assume now that *p*' ∈ ℝ*^m ^*is also a solution for (48), but $p_{c}^{\prime }\neq 0$ for some *c *∈ ℤ, *l *+ 1 ≤ *c *≤ *m*. Then *p*' = *p *+ *v*, where Ȳ⋅v=0, and *v_c _*≠ 0. In this case, *p*″ = *p *+ *λ · v *is also a solution for (48) for any *λ *∈ ℝ and *λ *can always be chosen so that $p_{j}^{\prime \prime }\neq 0$ for *j *= 1, ..., *l*, and there is at least one index *l *+ 1 ≤ *c *≤ *m *for which $p_{c}^{\prime \prime }\neq 0$. However, this contradicts to the assumption that *p *is a dense solution for (48).

*Proof of ***P2**. This is a straightforward consequence of **P1**, since the unweighted directed graphs of all constrained dense realizations must be identical.

*Proof of ***P3**. If the graph structure of the constrained realization is unique, then it trivially implies that the structures of the constrained dense and sparse realizations are identical, since there exists only one possible constrained reaction structure. If the structures of the constrained dense and sparse realizations are identical, then the number of nonzero reaction rates is the same in any constrained realizations including the constrained dense ones. Then it follows from **P1 **that the constrained reaction structure is unique.

## Supplementary Material

Additional file 1 **Detailed numerical data of the CRNs shown in Examples 1-3**. This file contains the detailed data (i.e. stoichiometric matrices and reaction rate coefficients) of the dynamically equivalent reaction networks studied in Examples 1,2 and 3. The individual sheets correspond to the different examples. Click here for file
